# Positive exchange-bias and giant vertical hysteretic shift in La_0.3_Sr_0.7_FeO_3_/SrRuO_3_ bilayers

**DOI:** 10.1038/srep04138

**Published:** 2014-02-26

**Authors:** Rakesh Rana, Parul Pandey, R. P. Singh, D. S. Rana

**Affiliations:** 1 Department of Physics, Indian Institute of Science Education and Research Bhopal, Govindpura, Bhopal-462023, India

## Abstract

The exchange-bias effects in the mosaic epitaxial bilayers of the itinerant ferromagnet (FM) SrRuO_3_ and the antiferromagnetic (AFM) charge-ordered La_0.3_Sr_0.7_FeO_3_ were investigated. An uncharacteristic low-field positive exchange bias, a cooling-field driven reversal of positive to negative exchange-bias and a layer thickness optimised unusual vertical magnetization shift were all novel facets of exchange bias realized for the first time in magnetic oxides. The successive magnetic training induces a transition from positive to negative exchange bias regime with changes in domain configurations. These observations are well corroborated by the hysteretic loop asymmetries which display the modifications in the AFM spin correlations. These exotic features emphasize the key role of i) mosaic disorder induced subtle interplay of competing AFM-superexchange and FM double exchange at the exchange biased interface and, ii) training induced irrecoverable alterations in the AFM spin structure.

The discovery of exchange bias (EB) effect by Meiklejohn and Bean^1^ has garnered enormous interest from the scientific community for its intriguing fundamental and technological aspects. Recent impetus on EB have resulted in diverse tantalizing avenues as the modern day electronic devices include its usage in spin valves, magnetic recording read heads, giant magnetoresistive sensors, etc^2^,^3^. The EB is usually characterized by an asymmetric shift in the magnetic hysteresis loop along the field axis when a ferromagnetic (FM)-antiferromagnetic (AFM) layered or a composite system is cooled in a static magnetic field through the Nèel temperature (T_N_) of the AFM phase^4^. The magnitude of the loop shift (H_EB_) depends on various factors such as the interfacial roughness, characteristics of the FM-AFM layers involved, the complex spin structure at the interface, the uncompensated moments at the interface, etc^4^,^5^,^6^. Usually for FM-AFM systems, the shift of the hysteresis loop is opposite to the cooling field (H_CF_) direction and is termed as negative exchange bias (NEB). On the other hand, the shift of hysteresis loop along the same sign of H_CF_ is termed as positive exchange bias (PEB)^5^,^6^. The PEB, a rarely observed phenomenon, was first reported for FeF_2_/Fe bilayer thin-films^5^,^6^. It is attributed to the AFM exchange coupling with its sign and magnitude strongly dependent on the H_CF_^5^,^6^. The AFM exchange coupling at the interface was also reported for two FM perovskite oxides, namely, La_2/3_Sr_1/3_MnO_3_ and SrRuO_3_^7^. The Cu_1-x_Mn_x_/Co bilayers exhibited PEB in the vicinity of blocking temperature which subsequently vanishes at lower temperatures resulting in NEB due to the coexistence of FM and AFM interface coupling^8^. More recently, the PEB for Ni_81_Fe_19_/Ir_20_Mn_80_ bilayers was observed and explained in the framework of meta-stable magnetic disorder at the FM-AFM interface induced by the magnetic training effect (TE)^9^.

Initially, most of the scientific quest to unravel the EB phenomenon was seen on metallic systems^1^,^3^,^4^,^5^,^6^,^7^,^8^,^9^,^10^,^11^. Recently, however, this phenomenon is also being explored and tuned in the magnetic perovskite oxides^7^,^12^,^13^,^14^,^15^,^16^. Understanding the evolution of EB in perovskites oxide bilayers and multilayers is essential as these systems present a greater degree of freedom for tunability of EB at the interface via strain, orbital reconstruction, charge-transfer, etc. Their suitable combinations with structural compatibility at the FM-AFM interface might unveil many potent facets of EB. Observation of EB in the disordered-ordered magnetic interfaces, *i.e.*, in paramagnetic (PM) LaNiO_3_ and FM LaMnO_3_ superlattice and the PM CaRuO_3_ and AFM CaMnO_3_ superlattices are clearly the recent important discoveries in this area^12^,^13^. More recently, strain engineered unexpected EB with the emergence of a self assembled spin glass like phase of LaSrMnO_4_ at the film/substrate interface was reported for (La,Sr)MnO_3_ single thin-films^17^. All endeavours are focussed on controlling and manipulation of EB by the interfacial interactions, thickness and number of layers of the FM and AFM phases, and the type of AFM order in the superlattice structures^14^,^15^. Overall the progress in EB has been two-fold. First, the EB has been addressed in unconventional heterostructures/bilayers with FM-PM, AFM-PM and collinear-noncollinear magnetic heterostructures^7^,^12^,^13^,^16^. This has challenged our present understanding of EB which is generally observed in conventional FM-AFM heterostructures^14^,^15^. The second focus has been to tune and realize the novel EB properties beyond NEB. For instance, the realization of PEB and its reversal to NEB with critical role played by both the extrinsic and the intrinsic factors in controlling PEB, are essential components yet to be explicitly realized and understood.

In this communication, we report a novel and unique set of EB properties in orthoferrtite-ruthenate bilayers La_0.3_Sr_0.7_FeO_3_/SrRuO_3_ (LSFO/SRO) fabricated on mosaic and non-mosaic SrTiO_3_ (STO) (111) substrate. These samples, henceforth will be referred to as LS_Mosaic_ and LS_Non-mosaic_, respectively. The proximity of the magnetic transition temperatures of the G-type AFM LSFO (T_N_ ~ 190 K) and the FM SRO (T_C_ ~ 160 K) makes them a suitable combination for investigation of EB properties in bilayer thin-film^18^,^19^,^20^,^21^. The (111) orientation of STO was chosen as it presents opportunity for increased interactions at the interface as compared to the conventional (100) STO substrate. This occurs as the [Fe^3+^/Fe^5+^] ions in the AFM LSFO will be surrounded by three of the same type and three of the other type *i.e.* Ru^4+^ ions of the FM SrRuO_3_^12^. We observe a low-field PEB, its sign reversal by both extrinsic and intrinsic factors and achieved a gigantic vertical magnetization shift. In this bilayer system, G-type AFM structure of LSFO coupled with FM SRO present an opportunity to control the EB by intriguing intrinsic factors such as nearest neighbour spin compensation, spin-flop coupling and competing superexchange (SE) interactions between FM and AFM resulting in a spin glass like interface. Whereas, the mosaicity of the substrate introduces external factors such as modulated spin structure at domain walls, random defects, and interface roughness to control and manipulate the EB. Formation of LSFO/SRO bilayers on both the mosaic and non-mosaic STO (111) substrate helps extract the contribution of extrinsic and intrinsic factors responsible for novel features of EB. A unique exhibition of diverse EB properties in LSFO/SRO observed here has been explained in the framework of modulation of the interfacial AFM spin structure with H_CF_ and training induced subsequent runs.

## Results

**Figure 1:**
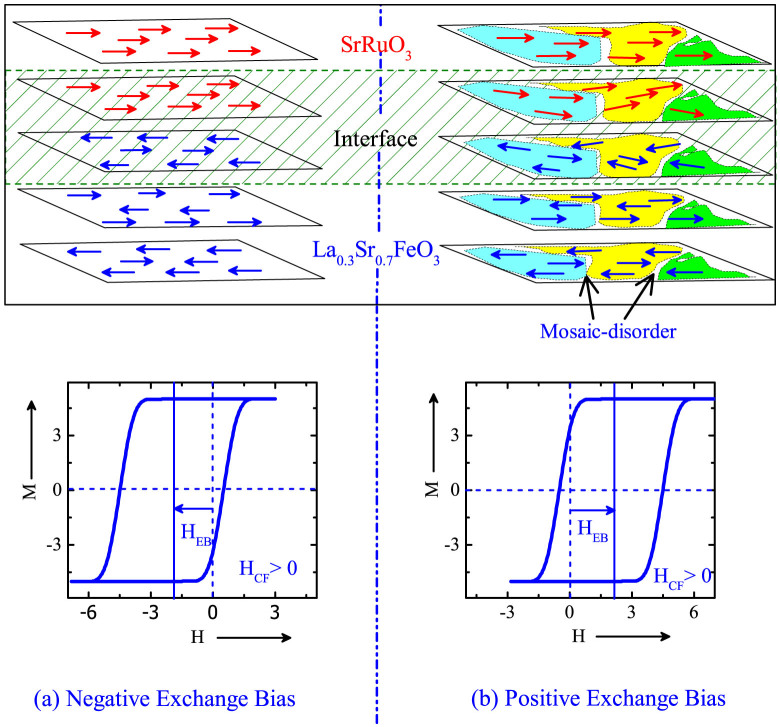
Schematic of an idealized illustration of the spins (arrows) for La_0.3_Sr_0.7_FeO_3_/SrRuO_3_ (AFM/FM) bilayer in, (a) an ordered interface on non-mosaic SrTiO_3_ substrate (LS_Non-mosaic_) and (b) disordered interface on the mosaic SrTiO_3_ substrate (LS_Mosaic_) [where, cooling field (H_CF_) is parallel to the film-plane].

**Figure 2:**
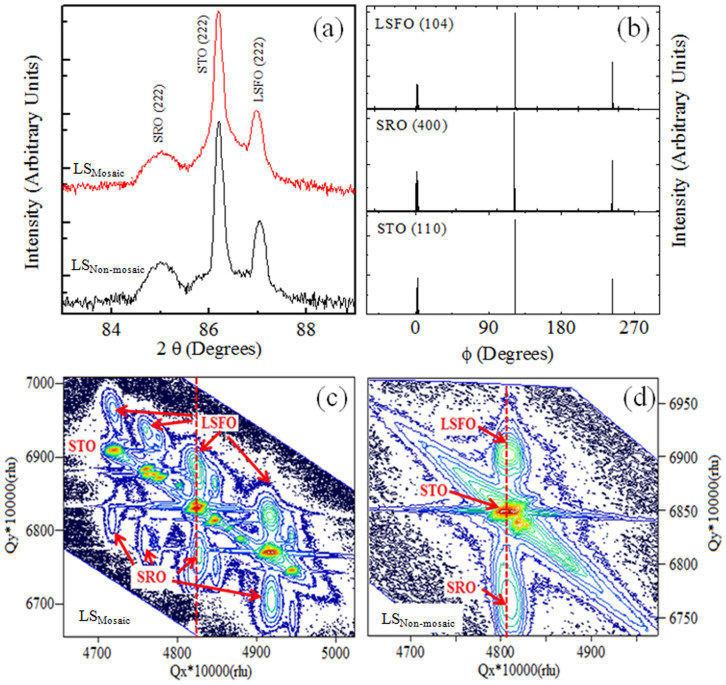
(a) shows *θ − 2θ* scan for LS_Mosaic_ and LS_Non-mosaic_ sample, (b) *ϕ*-scans along the peaks (104) for LSFO, (400) for SRO and (110) for STO substrate, (c–d) shows the reciprocal space maps for LS_Mosaic_ and LS_Non-mosaic_ along the asymmetric (330) orientation of the mosaic and non-mosaic STO (111) substrate, respectively.

A simplified illustration of the spins at the interface in the LSFO/SRO (FM/AFM) bilayers is shown in figure 1. The ordered and the disordered interfaces typically arise from the non-mosaic and the mosaic STO substrates, respectively [figure 1]. The *θ* − *2θ* XRD scans confirmed the phase purity of LS_Mosaic_ and LS_Non-mosaic_ samples [figure 2(a)]. In-plane epitaxial relationship was established by extracting the azimuthal-*ϕ* scans along the various peaks, *i.e*. (104) for LSFO, (400) for SRO and (110) for STO in the LS_Mosaic_ [figure 2(b)]. Three peaks in *ϕ*-scans with a separation of 120 degrees are observed for LSFO, SRO and STO which is expected to arise from the three fold symmetry of the STO (111) substrate. The mosaicity of the LS_Mosaic_ is distinctly evident in the reciprocal space map (RSM) scans around the asymmetric (330) peak. It shows that the STO substrate peak is split into multiple spots [figure 2(c) and supplementary S1]. This typically depicts that the substrate surface consists of several small crystalline blocks and each block corresponds to one of the reflection of the substrate in the RSM map as shown in figure 2(c). Further, corresponding to each substrate reflection there exists a reflection of the coherently strained LSFO and SRO epitaxial layers for the LS_Mosaic_. Such exhibition of multiple epitaxial peaks is absent in the LS_Non-mosaic_ sample which is formed on non-mosaic STO substrate [figure 2(d)]. The bulk pseudo-cubic lattice parameter of the LSFO is 3.87 Å, SRO is 3.93 Å and the STO is 3.905 Å. The out-of-plane lattice constant for the LSFO is 3.85 Å and the SRO is 3.945 Å. This suggests that the LSFO is under tensile strain, whereas, the SRO is under compressive strain. Overall, we can recognize qualitatively different crystal structures of the same substrate on which the LSFO/SRO bilayers namely, LS_Mosaic_ and LS_Non-mosaic_, were fabricated and their respective implications on the EB properties studied.

**Figure 3:**
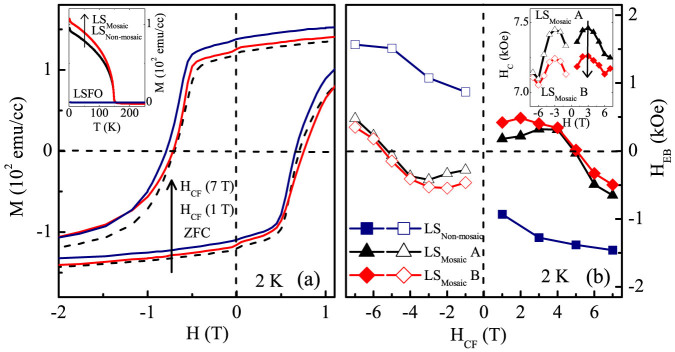
(a) Magnetization (M) versus magnetic field (H) loops of LS_Mosaic_ in zero field cooling (ZFC) and at various cooling fields (H_CF_), inset shows M versus temperature (T) plot in field cool warming protocol (H = 500 Oe) for LS_Mosaic_, LS_Non-mosaic_ and LSFO and (b) shows H_CF_ dependence of exchange bias (H_EB_) for LS_Mosaic_ and LS_Non-mosaic_ sample, inset depicts the training induced decrease in coercivity (H_C_) of LS_Mosaic_.

Magnetization (M) versus temperature (T) at a magnetic field (H) of 500 Oe in the field cooled cooling (FCC) protocol shows a T_C_ ~ 150 K for LS_Mosaic_ and LS_Non-mosaic_ [inset figure 3(a)]. This is slightly lower than the bulk T_C_ ~ 160 K of the SRO, presumably, due to strain in the thin film^14^,^15^. The M versus H loops at 2 K for zero-field cooling (ZFC) and in different H_CF_ for LS_Mosaic_ are shown in figure 3(a). It may be seen that the M-H loops for LS_Mosaic_ exhibits dissimilar manifestation of the H_EB_ with H_CF_. On one hand, we observe PEB for LS_Mosaic_ at low cooling field (H_CF_) ~ 1 T [>Hc] while, on the other hand, a H_CF_ of ~7 T dramatically supplants this PEB to a NEB regime [figure 3(a)]. This, in essence, is displayed in figure 3(b), where an unusual crossover from PEB to NEB ~5 T is observed. In contrast to this the LS_Non-mosaic_ sample exhibits only NEB at various H_CF_ which saturates in a field of ~5 T [figure 3(b)]. Overall, the EB properties of LS_Mosaic_ are novel and unusual, whereas, the EB for LS_Non-mosaic_ is rather conventional and is commonly observed for FM-AFM systems.

In the LS_Mosaic_ sample the mosaicity of the substrate induces topographic modulations which results in randomly oriented AFM easy axis of AFM grains in LSFO layer with a FM SRO layer coupled on to it. These sporadic distributions of magnetic inhomogeneities, having imperfections and defects at the interface result in various spin frustrated ensembles with a mixture of FM, AFM and spin flop coupling regimes^22^,^23^. The resultant of these microscopic FM-AFM exchange interactions at the interface and at the grain boundaries is understood to govern the dynamics of the system. The H_CF_ drives the LS_Mosaic_ in two ways, namely, i) at low H_CF_ [H_C_ < H_CF_ < 5 T], the microscopic AFM superexchange (AFM-SE) interactions dominate the FM double exchange at the interface and result in the PEB [figure 2(a)] and ii) as the H_CF_ is increased above 5 T, FM double exchange gets strengthened and dominates the microscopic AFM exchange at the interface giving NEB. Thus, a PEB → NEB crossover can be tuned via subtle interplay of surface AFM spin correlations with H_CF_.

**Figure 4:**
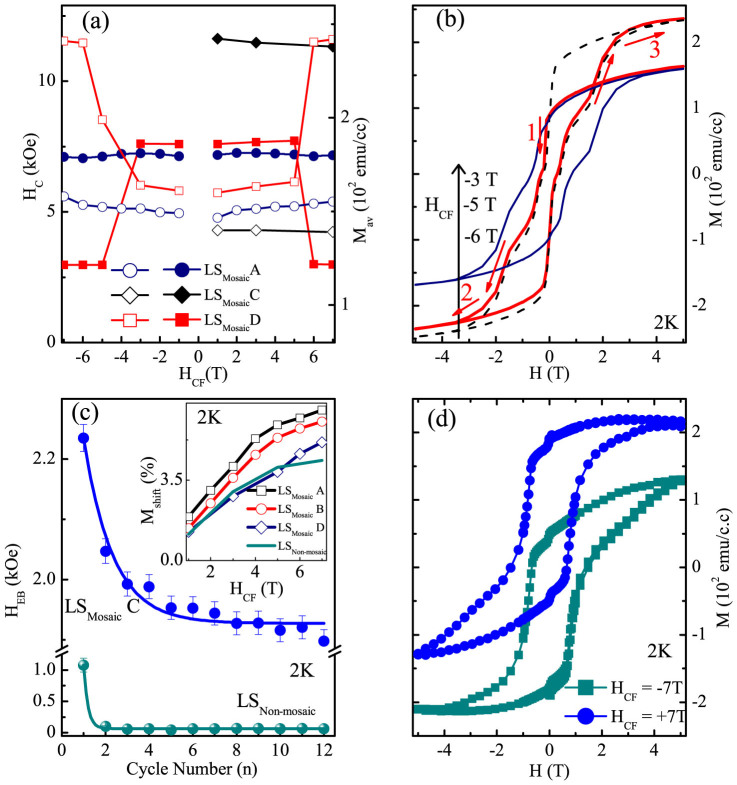
(a) Coercivity (H_C_) (closed symbols) and average saturation magnetization (M_av_) (open symbols) versus cooling field (H_CF_) at a temperature of 2 K, (b) Magnetization (M) versus magnetic field (H) loops at different H_CF_ for LS_Mosaic_D, (c) Exchange bias (H_EB_) with number of cycles (n) [solid line is the fit as per equation. 1] for LS_Mosaic_C and LS_Non-mosaic_, inset shows vertical shift (M_shift_) versus H_CF_ for LS_Mosaic_ (LS_Mosaic_A → LS_Mosaic_D) and LS_Non-mosaic_ samples, and (d) shows the maximum M_shift_ (~35%) for the optimized bilayer [LSFO (110 nm)/SRO(10 nm)].

To gain deeper insight of AFM spin correlations, we performed a multistage training cycles on the LS_Mosaic_ and the LS_Non-mosaic_ sample^24^,^25^,^26^. This was experimentally realized in the following sequence; LS_Mosaic_A (initial cycle) → LS_Mosaic_B (after 15 cycles) → LS_Mosaic_C (after 15cycles) → LS_Mosaic_D (after 12 cycles), while for nonmosaic LS_Non-mosaic_ (12 cycles) [1 cycle is the loop recorded at 2 K with H_CF_ = +7 T]. Training from LS_Mosaic_A to LS_Mosaic_B, causes a marginal increase in the PEB with a slight decrease in H_C_ [inset figure 3(b)]. Further, training results in vanishing of the PEB with a complete emergence of NEB regime for LS_Mosaic_C [figure 4(c)]. This NEB for the LS_Mosaic_C is associated with an increased H_C_ and a decreased M_av_ [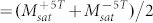] compared to that for LS_Mosaic_A [figure 4(a)] suggesting enhanced spin-flop coupling for LS_Mosaic_C^16^. The subsequent training cycle yields to LS_Mosaic_D, which shows a transition in shape of the hysteresis loop as a function of H_CF_ at 2 K [figure 4(b)]. It may be seen for LS_Mosaic_D the H_CF_ of −3 T yields a NEB loop [figure 4(b)]. As this H_CF_ is increased to −5 T the loop manifests with a lesser H_C_ [step1 to 2] with a marked increase in overall M [step 2–3]. Another loop recorded with H_CF_ of −6 T displays an entirely different shape as switching field (H_C_) decreases, as compared to the loop recorded with H_CF_ of −3 T [figure 4(b)]. This indicates that the pinning defects in the AFM layer are undergoing changes not only with training runs but have H_CF_ sensitivity as well.

The disorder induced in the LS_Mosaic_ is quite intriguing, as training causes H_EB_ to traverse from PEB (LS_Mosai_A–B) to NEB (LS_Mosaic_C–D) regime, whereas its counterpart LS_Non-mosaic_ exhibits NEB regime only. The TE is essential signature and can unveil the microstructural spin rearrangements along with the possible mechanisms driving the H_EB_. To understand the underlying intricacies, we compared the influence of training in the NEB regime of LS_Mosaic_ C with that of the LS_Non-mosaic_. The training leads to irreversible changes in the interfacial domain configurations, which causes the magnetization of the LSFO pinning layer to be nonconserved^26^. Such relaxation effects in the nonconserved order parameters can be addressed using Landau-Khalatnikov expression which was successfully employed to describe the TE in LSMO/SRO heterostructures^26^. The phenomenological expression used to model the cycle dependence (n) with H_EB_ is, 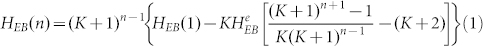
where, K and 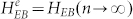 are the crucial fitting parameters, *H_EB_*(1) is the first loop H_EB_ value. The equation (1) can also be written as 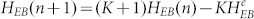
26. The value of K usually lies in the range −1 ≤ *K* ≤ 0^26^. When K = 0, it yields *H_EB_*(*n* + 1) = *H_EB_*(*n*) implying no training, whereas for K = −1, it is 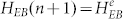 which yields a step like change in H_EB_ between the first two data points with no TE for *n* > 2^26^. Equation (1) was successfully fitted to both LS_Mosaic_C and LS_Non-mosaic_, with the values of K as −0.52 and −0.97, respectively. For *n* ≥ 2, the H_EB_ for LS_Mosaic_C keeps on decreasing with *n*, whereas, the H_EB_ for LS_Non-mosaic_ exhibits a negligible change.

The contrasting training behaviour for LS_Mosaic_C and LS_Non-mosaic_, plausibly indicates different training mechanisms governing both the samples. We attribute the initial large decrease in H_EB_ for both the samples to a ‘Hoffmann’ like behaviour, where the major changes after the first reversal can be ascribed to a transformation from an initial noncollinear arrangement of the AFM spins to a more relaxed collinear arrangement^27^. Furthermore, as per Hoffmann's model, the TE should cease for *n* ≥ 2^27^. This is displayed by LS_Non-mosaic_, whereas, LS_Mosaic_C shows a continuous decrease in H_EB_ even beyond *n* ≥ 2. This decrease in H_EB_ (*n* ≥ 2) for LS_Mosaic_, typically indicates that along with the Hoffman's component (which largely trains out after the first cycle), a second contribution to training may be present. This seems to arise from the thermally activated depinning of the uncompensated AFM spins^28^,^29^. Thus, the LS_Mosaic_ and the LS_Non-mosaic_ can explicitly be distinguished via field training, as the former exhibits a combination of a Hoffman and thermally activated depinning mechanism, whereas, the later trains out via ‘Hoffman’ mechanism^27^,^28^.

We also observed a positive vertical magnetization shift in the hysteresis loop along the same sign as of the H_CF_ for both the samples LS_Mosaic_ and LS_Non-mosaic_ [inset figure 4(c)]. Interestingly, vertical shift also displays the TE as it decreases from LS_Mosaic_A → LS_Mosaic_D [inset figure 4 (c)]. Vertical shift can be calculated using, 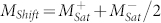, where, 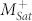 and 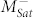 are positive and negative saturation values of the hysteresis loop. Observation of vertical shift is rare and usually points towards the uncompensated spins at the FM-AFM interface or that are in the bulk AFM^14^,^15^,^30^,^31^,^32^. Further, this rare and intriguing observation of vertical shift present in our bilayer system on STO (111) was found to vary with the thickness of AFM LSFO layer [unpublished data]. Thickness variation in AFM or FM phase of a FM/AFM bilayer system is an essential component to control the H_EB_, H_C_ and can also be used to tune the vertical shift^33^,^34^,^35^. We noted a maximum vertical shift of 35% for our optimized bilayer sample with LSFO(110 nm)/SRO(10 nm) on non-mosaic STO(111) [figure 4(d)].

**Figure 5:**
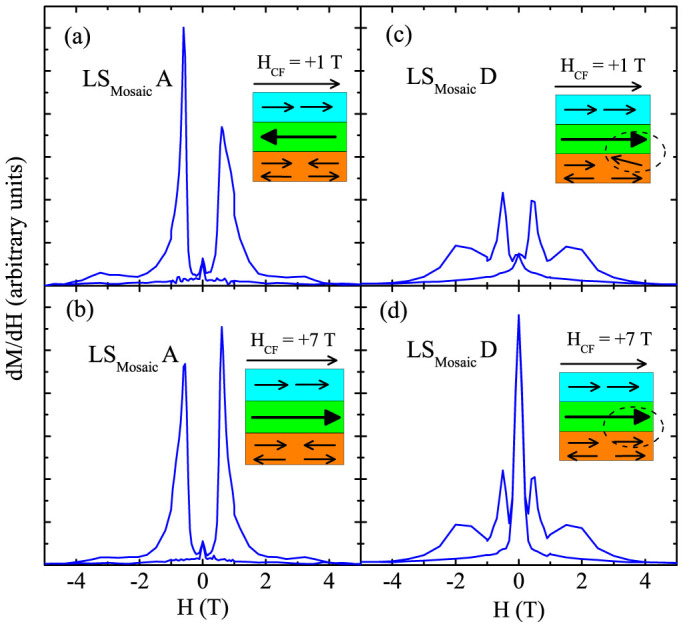
Asymmetry in hysteresis loop (dM/dH) versus magnetic field (H) for LS_Mosaic_A and LS_Mosaic_D at different cooling field (H_CF_). Inset boxes with orange, green and blue colour depicts the spin configurations of La_0.3_Sr_0.7_FeO_3_/Interface/SrRuO_3_, respectively.

For further analysis of the sign reversal of the EB of LS_Mosaic_, the loop asymmetries (dM/dH) were derived from the hysteresis data and are shown in figure 5^9^. It may be seen that for low positive H_CF_ (1 T) the first loop reversal is sharper than the second reversal of the loop [figure 5(a)] and yields PEB. As the H_CF_ is increased to +7 T the peak height is reversed and yields a transformation to a NEB regime for the LS_Mosaic_A [figure 5(b)]. This shows the sensitivity of the AFM spin structure to the H_CF_ and points towards a change in the microscopic AFM to FM exchange interaction at the interface [see schematic in figure 5(a) (AFM interface coupling) → 5 (b) (FM interface coupling)]. The shape of the subsequent hysteresis loops after training is more symmetric and rounded for LS_Mosaic_C [not shown and is similar to figure 5(c)]. Furthermore, a peak in the vicinity of H = 0 T for LS_Mosaic_D [figure 5(d)] shows that the FM spins have now softened and are very sensitive to any reversal of the direction of sweeping field. This scenario is in good congruence with that discussed earlier for figure 4 (b) in which we observed an enhanced saturation M with a decreased H_C_. The loop asymmetries as described above portrays the significant deviations in the pinning AFM layer with the H_CF_ and training runs resulting in PEB → NEB transition [Inset figure 5(a–d)].

**Figure 6:**
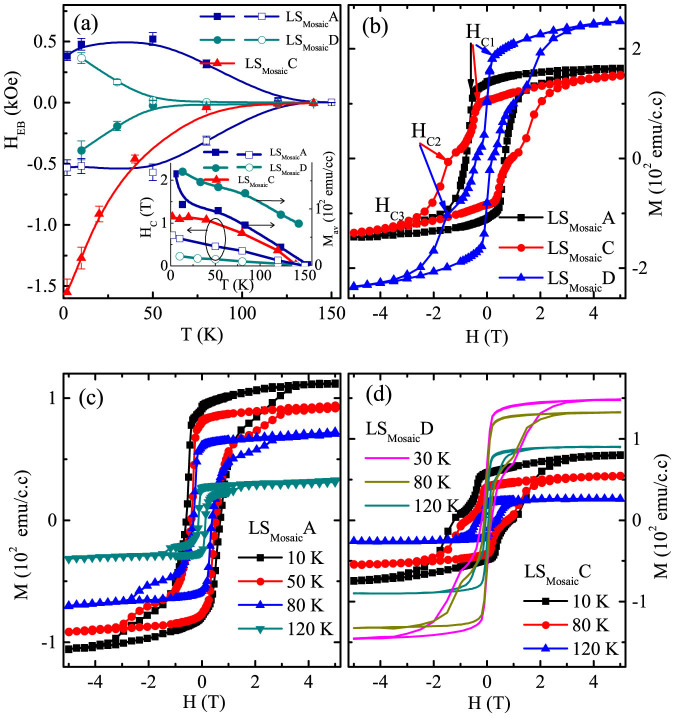
(a) Exchange Bias (H_EB_) versus temperature (T) for LS_Mosaic_ at a cooling field of +3 T (solid symbols) and −3 T (hollow symbols), dashed line is fit as per equation 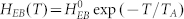, while the solid line is guide to the eye. Inset depicts temperature variation of H_C_ and M_av_ for LS_Mosaic_ sample, (b), (c) & (d) shows the temperature variation of hysteresis loop shapes for LS_Mosaic_A, LS_Mosaic_C and LS_Mosaic_D.

Figure 6(a) illustrates the temperature dependence of the H_EB_ for the LS_Mosaic_ sample after various training runs. The blocking temperature for LS_Mosaic and_ LS_Non-mosaic_ is nearly the same 130 K [Supplementary figure S2]. We find that for LS_Mosaic_A exhibiting PEB, the H_EB_ increases slightly for a temperature upto 50 K and then it shows a decrease with increasing temperature [Figure 6(a)]. In the NEB regime for LS_Mosaic_C and LS_Mosaic_D the H_EB_ exhibits an exponential type of decrease with increasing temperature. This usually signifies the frustrated spin state at the interface^36^,^37^. To substantiate this the H_EB_ data of LS_Mosaic_C and LS_Mosaic_D were fitted to the equation 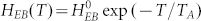, where 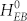 is the extrapolation of *H_EB_* at absolute zero and *T_A_* is a constant [figure 6 (a)]^36^,^37^. We obtained convincing fits with, 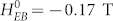 and −0.063 T with *T_A_* = 30 K and 21 K for LS_Mosaic_C and LS_Mosaic_D, respectively. Further, inset figure 6(a) depicts the temperature variation in the H_C_ and M_av_ for LS_Mosaic_ sample. We observed an enhanced overall M_av_ for LS_Mosaic_D, as compared to that of LS_Mosaic_(A–C) in the entire temperature range [figure 6(b,c and d)]. This suggests that the training causes a temperature independent retention of the irrecoverable permanent spin rearrangements in the AFM layer for the LS_Mosaic_D.

## Discussions

In this section we will discuss the key observations of the LS_Mosaic_ sample, in the following sequence, i) competing exchange interactions at the LSFO/SRO interface and the possible EB model for the observed PEB, ii) dynamics of the training induced dissimilar hysteresis loop shape transitions, and iii) the vertical magnetization shift.

The subtle interplay of FM-SE and AFM-SE interactions at the LSFO/SRO interface drives the PEB → NEB transition in the LS_Mosaic_ sample. The transition may be attributed to a potential crossover from AFM to FM exchange coupling [figure 3(b)]. This occurs as the mosaicity induces a disorder at the LS_mosaic_ interface, thus, inducing the competition between FM-AFM exchange interactions. On one hand, LSFO grain boundaries exhibit FM-SE interaction in Fe^5+^-O-Fe^3+^ and AFM-SE interaction in Fe^3+^-O-Fe^3+^ in the [001] plane^20^. On the other hand, across the FM-AFM interface Ru^4+^-O-Fe^3+^ and Ru^4+^-O-Fe^5+^ exhibits a FM double exchange interaction. The increasing H_CF_ overcomes the localized AFM-SE interaction and strengthens the FM double exchange resulting in a crossover from PEB to NEB regime. Several models were proposed to explain the EB effect^22^,^23^,^38^,^39^,^40^,^41^,^42^,^43^,^44^,^45^. The EB in mosaic LS_Mosaic_ sample is suggestive of a scenario in which the interface domain wall (IDW) develops as a result of competition between AFM coupling and the Zeeman energy^44^,^45^. Presently, IDW can manifest between different crystallite ensembles, consisting of independent AFM grain boundaries with a coupled FM layer on to it. The IDW can provide AFM coupling at the interface which will yield PEB for LS_Mosaic_A. Also, IDW shows training and H_CF_ sensitivity. Thus, as the H_CF_ is increased thickness of IDW may decrease due to domain wall compression, yielding a complete NEB regime for LS_Mosaic_ C–D^44^,^45^.

At this point, it is imperative to discuss the possibility of charge transfer at the LSFO-SRO interface. Charge transfer was found to be associated with the observed unidirectional anisotropy in LSMO/YBCO^46^,^47^. In contrast, for the La_2_CuO_4_/LSMO bilayers, it was demonstrated that charge transfer is not a key factor, as the H_C_ was found to exhibit a AFM thickness dependence [keeping FM thickness constant]. In the present case too, the H_C_ was found to vary with the LSFO thickness for the LSFO/SRO bilayers on nonmosaic STO substrate [unpublished data]. This further bolsters the dominant role of SE interaction at the LSFO/SRO spin-glass like interface.

The TE introduces irreversible changes in the LSFO layer and at the LSFO/SRO interface, which manifests in the form of a magnetic reorientation from a square loop [LS1A–B] to a stepped hysteresis loop [LS1C–D] [figure 6 (b)]^48^. Interestingly, this loop shape variation may be associated with an enhancement in spin-flop coupling strength (*J_ex_*). For the LS_Mosaic _sample, the strength of spin-flop coupling at the interface can be estimated using, *J_ex_* = *H_EB_t_FM_M_S_*, (where, *t_FM_* is thickness of FM SRO layer, and *M_S_* is saturation magnetization)^48^. The deduced value of *J_ex_* (2 K) for LS_Mosaic_(A–B) → LS_Mosaic_C → LS_Mosaic_D varies as 0.2 → 0.66 → 0.57 erg/cm^2^. Apparently higher value of *J_ex_* substantiates the enhanced spin-flop coupling in LS_Mosaic_C–D which yields a stepped hysteresis loop, whereas a low *J_ex_* favours a square loop in LS_Mosaic_A–B.

Now, we further discuss the implications of the multistage training runs and switching of the hysteresis loops in the LS_Mosaic_ sample [figure 6 (b)]^49^,^50^,^51^. The LS_Mosaic_A sample exhibits a coherent reversal of the hysteresis loop in the whole temperature range [figure 6(c)]. On the other hand, this coherent reversal of the SRO spins is hindered at H_C2_ for the LS_Mosaic_C–D and the loop closes at H_C3_. This emergence of H_C2_ can be associated with the domain wall depinning processes which may be training or thermally assisted^28^,^29^,^50^,^51^. Further the TE largely alters the pinned spin concentration from LS_Mosaic_A to LS_Mosaic_D. This is evident as the relative changes in H_C1_ with temperature are quite pronounced for LS_Mosaic_A and LS_Mosaic_C. In contrast, the LS_Mosaic_D exhibits a negligible change in H_C1_. This indicates that the pinning defects concentration have been drastically reduced for LS_Mosaic_D with subsequent training runs. Furthermore, the H_C1_ was found to decrease from −0.4 T (LS_Mosaic_C) to +0.1 T (LS_Mosaic_D). This points towards a sharp reversal of the SRO spins even before H = 0. Remarkably, this was also evident in the loop asymmetries, as a sharp peak was observed near H = 0 [figure 5(d)]. The nearly temperature independent trend of H_C1_ for LS_Mosaic_D suggests that the LSFO interfacial spins have now been depinned and have started reversing with the FM SRO spins. This causes drastic reduction in H_C_ for LS_Mosaic_D, which is also accompanied with a huge increase (64%) in M_av_ of the loops [figure 3(b)]. This excess M in LS_Mosaic_D may have contributions from, i) the interfacial AFM ions Fe^5+^ (~1.5 *μ_B_*) and Fe^3+^ (~3.5 *μ_B_*) which have started rotating coherently with the FM layer^20^, ii) the, FM SRO might break into mixture of different regions (hard and soft), for large H_C_ hard regions out number their softer counterparts and vice versa^52^.

Finally, we comment on another important observation, which is the vertical magnetization shift [inset figures 4(c) and 4(d)]. The observation of vertical shift along the same sign as of the H_CF_ usually indicates FM coupling at the interface^5^,^6^. We observed a positive vertical shift for LS_Mosaic_A and LS_Non-mosaic_ which suggests FM coupling at interface. But, interestingly, LS_Mosaic_A also exhibits a PEB, which point towards the AFM coupling at the interface. Nevertheless, similar contrasting scenario was well addressed by Fritzimmons *et al.*, as they showed that a microscopic AFM coupling at the interface is likely possible and can manifest along with a positive vertical shift^30^. This is seen for LS_Mosaic_A sample. Moreover, a giant vertical shift of about 35% for our optimized sample suggests that a large number of uncompensated AFM spins exists when the bilayer is grown along (111) orientation of STO [figure 4(d)]. This may occur as LSFO is known to exhibit an intriguing quasi-2D charge ordering on STO (111) rather than a perfect 3D charge ordered regime with a charge-disproportionate Fe^3+^ and Fe^5+^ ions along (111)^19^. The latent defects and imperfections in the film may give rise to uncompensated spins in the bulk along with the surface AFM spins resulting in massive EB.

To summarize, we report a novel method of mosaicity induced disorder to obtain a rare phenomenon of PEB, magnetic annealing and H_CF_ induce PEB → NEB transition and accompanying loop shape transitions. While the mosaic-disorder induces AFM exchange coupling at the interface which causes PEB, the uncompensated spins arising from the intrinsic nature of the magnetic order of LSFO yield the huge vertical shift. These studies open up new avenues for obtaining the otherwise elusive PEB for FM/AFM systems and an innovative way to tune giant vertical shift in magnetic oxides.

## Methods

The bilayers of LSFO as bottom layer and SRO as top layer were fabricated on STO (111) single crystal substrates by pulsed laser deposition (PLD) technique using a 248 nm KrF excimer laser. Deposition was carried out at a repetition rate of 4 Hz with laser energy of 1.7 J/cm^2^ at the target with a substrate temperature of 700°C, oxygen partial pressure of 25 Pa and a post-deposition annealing for 5 minutes in 1.5 kPa of O_2_. Thickness of the bilayers with LSFO (37 nm) and SRO (20 nm) for LS_Mosaic_ and LS_Non-mosaic_ were measured using a surface profiler. The X-ray diffraction (XRD) measurements were carried out using PANalytical Empyrean. Magnetization measurements were performed on a SQUID magnetometer (Quantum design, USA).

## Author Contributions

R.R. and D.S.R. conceived and designed the experiments. R.R. and P.P. carried out the experiments. R.R. and D.S.R. wrote the paper. R.R., P.P., R.P.S. and D.S.R. discussed the results and commented on the manuscript.

## Supplementary Material

Supplementary Information Positive exchange-bias and giant vertical hysteretic shift in La_0.3_Sr_0.7_FeO_3_/SrRuO_3_ bilayers

## References

[b1] Meiklejohn W. H. & Bean C. P. New magnetic anisotropy. Phys. Rev. 102, 1413 (1956).

[b2] Bibes M., Villegas J. E. & Barthelemy A. Ultrathin oxide films and interfaces for electronics and spintronics. Adv. Phys. 60, 5 (2011).

[b3] Dieny B. *et al.* Giant magnetoresistive in soft ferromagnetic multilayers. Phys. Rev. B 43, 1297 (1991). 10.1103/physrevb.43.1297 9996352

[b4] Nogués J. *et al.* Exchange bias in nanostructures. Phys. Rep. 422, 65 (2005).

[b5] Nogués J., Lederman D., Moran T. J. & Schuller I. K. Positive exchange bias in FeF_2_-Fe bilayers. Phys. Rev. Lett. 76, 4624 (1996). 10.1103/PhysRevLett.76.4624 10061338

[b6] Nogués J., Morellon L., Leighton C., Ibarra M. R. & Schuller I. K. Antiferromagnetic spin flop and exchange bias. Phys. Rev. B 61, R6455 (2000).

[b7] Ke X., Rzchowski M. S., Belenky L. J. & Eom C. B. Positive exchange bias in ferromagnetic La_0.67_Sr_0.33_MnO_3_/SrRuO_3_ bilayers. Appl. Phys. Lett. 84, 5458 (2004).

[b8] Ali M. *et al.* Exchange bias using a spin glass. Nature Mater. 6, 70–75 (2007). 10.1038/nmat1809 17173030

[b9] Mishra S. K., Radu F., Dürr H. A. & Eberhardt W. Training-induced positive exchange bias in NiFe/IrMn bilayers. Phys. Rev. Lett. 102, 177208 (2009). 10.1103/PhysRevLett.102.177208 19518827

[b10] Malozemoff A. P. Mechanisms of exchange anisotropy (invited). J. Appl. Phys. 63, 3874 (1988).

[b11] Leighton C., Nogués J., Jönsson-Åkerman B. J. & Schuller I. K. Coercivity Enhancement in exchange biased systems driven by interfacial magnetic frustration. Phys. Rev. Lett. 84, 3466 (2000). 10.1103/PhysRevLett.84.3466 11019116

[b12] Gibert M., Zuboko P., Scherwitzl R., Íñiguez J. & Triscone J. M. Exchange bias in LaNiO_3_–LaMnO_3_ superlattices. Nature Mater. 11, 195–198 (2012). 10.1038/nmat3224 22266467

[b13] He C. *et al.* Interfacial ferromagnetism and exchange bias in CaRuO_3_/CaMnO_3_ Superlattices. Phys. Rev. Lett. 109, 197202 (2012). 10.1103/PhysRevLett.109.197202 23215420

[b14] Padhan P. & Prellier W. Coercivity enhancement in the SrRuO_3_/SrMnO_3_ superlattices. Appl. Phys. Lett. 88, 263114 (2006).

[b15] Choi Y. *et al.* Ferromagnetic Mn moments at SrRuO_3_/SrMnO_3_ interface. Appl. Phys. Lett. 91, 022503 (2007).

[b16] Tian Y. F. *et al.* Anomalous exchange bias at collinear/noncollinear spin interface. Sci. Rep. 3, 1094 (2013).

[b17] Cui B. *et al.* Strain engineering induced interfacial self-assembly and intrinsic exchange bias in a manganite perovskite film. Sci. Rep. 3, 2542 (2013). 10.1038/srep02542 PMC3756339 23985971

[b18] Li J. Q., Matsui Y., Park S. K. & Tokura Y. Charge ordered states in L_a1-x_S_rx_Fe_O3_. Phys. Rev. Lett. 79, 297 (1997).

[b19] Okamoto J. *et al.* Quasi-two-dimensional d-spin and p-hole ordering in the three-dimensional perovskite La_1/3_Sr_2/3_FeO_3_. Phys. Rev. B 82, 132402 (2010).

[b20] Sabyasachi S. *et al.* Glassy magnetic phase driven by short-range charge and magnetic ordering in nanocrystalline La_1/3_Sr_2/3_FeO_3 − δ_ Magnetization, Mössbauer, and polarized neutron studies. Phys. Rev. B 86, 104416 (2012).

[b21] Koster G. *et al.* Structure, physical properties, and applications of SrRuO_3_ thin films. Rev. Mod. Phys. 84, 253 (2012).

[b22] Koon N. C. Calculation of exchange bias in thin-films with ferromagnetic/antiferromagnetic interfaces. Phys. Rev. Lett. 78, 4865 (1997).

[b23] Schulthess T. C. & Butler W. H. Consequences of spin-flop coupling in exchange biased films. Phys. Rev. Lett. 81, 4516 (1998).

[b24] Binek C. Training of the exchange- bias effect: A simple analytic approach. Phys. Rev. B 70, 014421 (2004).

[b25] Binek C. h., Polisetty S., He & Berger A. Exchange bias training effect in coupled all ferromagnetic bilayer structures. Phys. Rev. Lett. 96, 067201 (2006). 10.1103/PhysRevLett.96.067201 16606037

[b26] Polisetty S., Sahoo S., Berger A. & Binek C. h. Temperature dependence of the training effect in exchange coupled ferromagnetic bilayers. Phys. Rev. B 78, 184426 (2008).

[b27] Hoffmann A. Symmetry driven irreversibilities at ferromagnetic-antiferromagnetic interfaces. Phys. Rev. Lett. 93, 097203 (2004). 10.1103/PhysRevLett.93.097203 15447135

[b28] Chan M. K., Parker J. S., Crowell P. A. & Leighton C. Identification and separation of two distinct contributions to the training effect in polycrystalline CoO/FeMn bilayers. Phys. Rev. B 77, 014420 (2008).

[b29] Stiles M. D. & McMichael R. D. Temperature dependence of exchange bias in polycrystalline ferromagnet-antiferromagnet bilayers. Phys. Rev. B 60, 12950 (1999).

[b30] Fitzsimmons M. R. *et al.* Pinned magnetization in the antiferromagnet and ferromagnet of an exchange bias system. Phys. Rev. B 75, 214412 (2007).

[b31] Gruyters M. & Schmitz D. Microscopic nature of ferro and antiferromagnetic interface coupling of uncompensated magnetic moments in Exchange Bias Systems. Phys. Rev. Lett. 100, 077205 (2008). 18352593 10.1103/PhysRevLett.100.077205

[b32] Ohldag H. *et al.* Correlation between exchange bias and pinned interfacial spins. Phys. Rev. Lett. 91, 017203 (2003). 10.1103/PhysRevLett.91.017203 12906569

[b33] Kobrinskii A. L., Goldman A. M., Varela M. & Pennycook S. J. Thickness dependence of the exchange bias in epitaxial manganite bilayers. Phys. Rev. B 79, 094405 (2009).

[b34] Binek C. h., Hochstrat A. & Kleemann W. Exchange bias in a generalized Meiklejohn-Bean approach. J. Magn. Magn. Mater. 234, 353 (2001).

[b35] Ding J. F., Tian Y. F., Hu W. J., Lin W. N. & Wu T. Exchange coupling and coercivity enhancement in cuprate/manganite bilayers. Appl. Phys. Lett. 102, 032401 (2013).

[b36] Moutis N., Christides C., Panagiotopoulos I. & Niarchos D. Exchange-coupling properties of La_1-x_Ca_x_MnO_3_ ferromagnetic/antiferromagnetic multilayers. Phys. Rev. B 64, 094429 (2001).

[b37] Ding J. F. *et al.* Interfacial spin glass state and exchange bias in manganite bilayers with competing magnetic orders. Phys. Rev. B 87, 054428 (2013).

[b38] Mauri D., Siegmann H. C., Bagus P. S. & Kay E. Simple model for thin ferromagnetic films exchange coupled to an antiferromagnetic substrate. J. Appl. Phys. 62, 3047 (1987).

[b39] Kiwi M., López J. M., Portugal R. D. & Ramírez R. Positive exchange bias model: Fe/FeF_2_ and Fe/MnF_2_ bilayers. Solid State Comm. 116, 315 (2000).

[b40] Stiles M. D. & McMichael R. D. Model for exchange bias in polycrystalline ferromagnet-antiferromagnet bilayers. Phys. Rev. B 59, 3722 (1999).

[b41] Nowak U. *et al.* Domain state model for exchange bias. I. Theory. Phys. Rev. B 66, 014430 (2002).

[b42] Keller J. *et al.* Domain state model for exchange bias. II. Experiments. Phys. Rev. B 66, 014431 (2002).

[b43] Dong S. *et al.* Exchange bias driven by the Dzyaloshinskii-Moriya interaction and ferroelectric polarization at G-type antiferromagnetic perovskite interfaces. Phys. Rev. Lett. 103, 127201 (2009). 10.1103/PhysRevLett.103.127201 19792455

[b44] Hauet T., Borchers J. A., Mangin P. h., Henry Y. & Mangin S. Training effect in an exchange bias system: the role of interfacial domain walls. Phys. Rev. Lett. 96, 067207 (2006). 10.1103/PhysRevLett.96.067207 16606043

[b45] Henry Y., Mangin S., Hauet T. & Montaigne F. Positive exchange-bias induced by interface domain wall quenching in GdFe/TbFe films. Phys. Rev. B 73, 134420 (2006).

[b46] Przyslupski P. *et al.* Magnetic properties of La_0.67_Sr_0.33_MnO_3_/YBa_2_Cu_3_O_7_ superlattices. Phys. Rev. B 69, 134428 (2004).

[b47] Holden T. *et al.* Proximity induced metal-insulator transition in YBa_2_Cu_3_O_7_/La_2/3_Ca_1/3_MnO_3_ superlattices. Phys. Rev. B 69, 064505 (2004).

[b48] Zhan Q. & Krishnan K. M. In-plane reorientation of magnetization in epitaxial exchange biased Fe/MnPd bilayers. Appl. Phys. Lett. 96, 112506 (2010).

[b49] Rothman J. *et al.* Observation of a bi-domain state and nucleation free switching in mesoscopic ring magnets. Phys. Rev. Lett. 86, 1098 (2001). 10.1103/PhysRevLett.86.1098 11178019

[b50] Kläui M. *et al.* Direct observation of spin configurations and classification of switching processes in mesoscopic ferromagnetic rings. Phys. Rev. B 68, 134426 (2003).

[b51] Kläui M. *et al.* Switching processes and switching reproducibility in ferromagnetic ring structures. Appl. Phys Lett. 84, 951 (2004).

[b52] Fullerton E. E., Jiang J. S., Grimsditch M., Sowers C. H. & Bader S. D. Exchange-spring behavior in epitaxial hard/soft magnetic bilayers. Phys. Rev. B 58, 12193 (1998).

