# Supplementary material for: Positive exchange-bias and giant vertical hysteretic shift in La0.3Sr0.7FeO3/SrRuO3 bilayers
Source: Sci Rep. 2014 Feb 26;4:4138. doi: 10.1038/srep04138 (PMC3935188; doi:10.1038/srep04138)
Supplement: Supplementary Information — Positive exchange-bias and giant vertical hysteretic shift in La0.3Sr0.7FeO3/SrRuO3 bilayers [file srep04138-s1.doc]

Supplementary Information

**Positive exchange-bias and giant vertical hysteretic shift in La0.3Sr0.7FeO3/SrRuO3 bilayers**

Rakesh Rana, Parul Pandey, R. P. Singh, D. S. Rana*

*Department of Physics, Indian Institute of Science Education and Research Bhopal, Govindpura, Bhopal-462023,*

*E-mail: [dsrana@iiserb.ac.in](mailto:dsrana@iiserb.ac.in)

**Figure S1:**


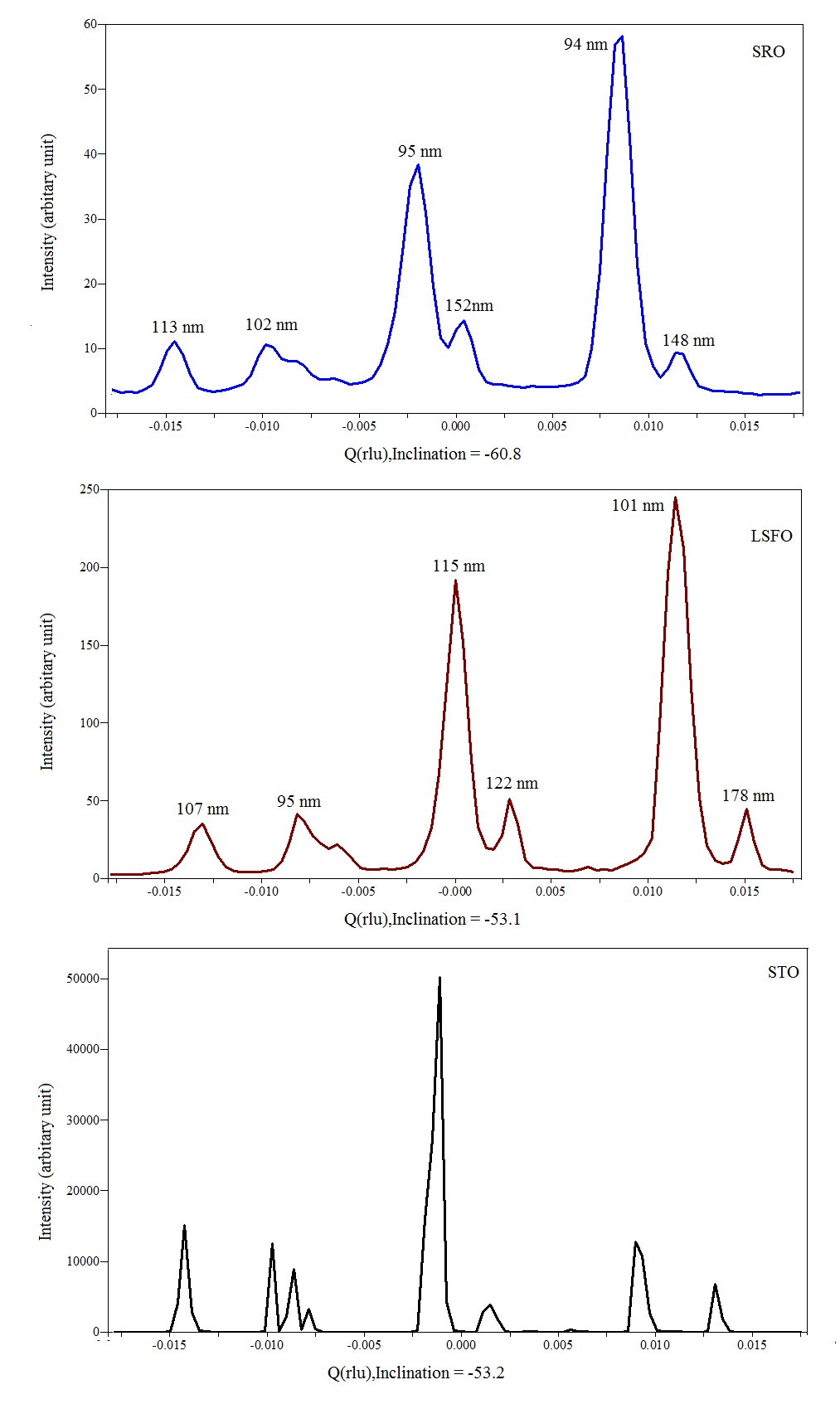


**Figure S1:** Q scans extracted from the asymmetric 330 reflection of the mosaic sample with different thickness grains was deduced using the Scherrer’s formula $\tau=k*\lambda/\beta*\cos(\theta)$ where$\tau$,$k,\lambda,\beta$and $\theta$ are thickness, sample dependent constant, wavelength of X-ray, FWHM (full width at half maximum of the peak) and Bragg’s angle respectively.

To characterize the mosaic grain distribution in the LSMosaic sample we have extracted the Q-scans from the asymmetric reflection (330) [figure. (S1)]. The Q-scans for La0.3Sr0.7FeO3 (LSFO) and SrRuO3 (SRO) display a broadening along the $\omega$direction with multiple peaks [figure S1]. Further, the grain size can be calculated using Scherrer’s formula for the LSFO and SRO layers [figure S1]. In-addition, the mosaicity can be characterized using the ‘*mosaic spread*’. It determines the misorientation of the perfect crystals within a given crystal. For the perfect crystals the mosaic spread is usually zero and for the mosaic ones it can have values as large as one degree. Mosaic spread is usually ascribed to the microscopic tilt in the sample, this may arise as a result of, i) a fine mosaic structure giving a variation in the tilt of the reflecting plane for different mosaic grains, or ii) the "rippled" reflecting planes due to the presence of a uniform dislocation density. This tilt range causes a broadening of the diffraction feature perpendicular to the line joining the reciprocal lattice point to the origin. The full width at half maximum (FWHM) in this perpendicular direction is used as a direct measure of the amount of mosaic spread present. Presently, for the LSMosaic sample the mosaic spread is $0.18^{o}$.

**Figure S2:**


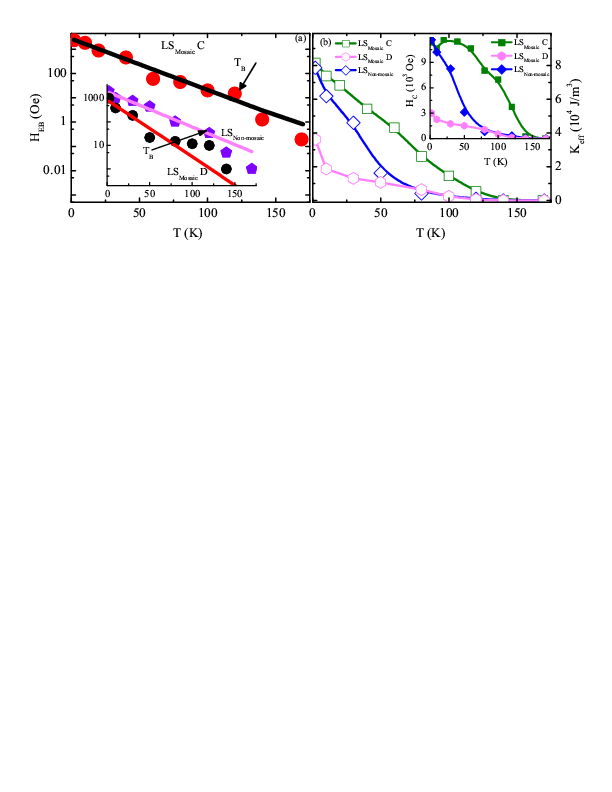


**Figure S2:** (a) Exchange-bias field (HEB) as a function of temperature (T) for LSMosaicC and LSMosaicD and LSNon-mosaic. Solid lines are exponential fits for HEB. (b) Temperature dependence of magnetic anisotropy Keff for LSMosaicC, LSMosaicD and LSNon-mosaic. Inset shows the coercivity (HC) as function of temperature for LSMosaicC, LSMosaicD and LSNon-mosaic.

The temperature (T) dependence of exchange bias (HEB) for the LSMosaicC, LSMosaicD and the LSNon-mosaic, is shown in figure S2 (a). The exponential fitting of HEB vs T in log scale for the LSMosaicC, LSMosaicD and the LSNon-mosaic shows a deviation from the linear behaviour at the blocking temperature (TB)­, and is depicted by the arrows in figure S2 (a).S1 TB is the temperature above which HEB vanishes. We can clearly see that the TB for the LSMosaicC and the LSNon-mosaic is at a temperature of ̴ 130 K. This is slightly less than the ferromagnetic ordering temperature (TC) of SrRuO3 (SRO) ̴ 150 K for both the films. Further, training alters the defect concentration from LSMosaicC to LSMosaicD, and this is also visible as the TB is lowered to a temperature of ̴ 80 K. This happens as training causes some of the surface LSFO spins to coherently rotate with the SRO spins which initially acted as the pinning sites.

Magnetic anisotropy, Keff, of versus temperature (T) for (LSMosaicC) and LSMosaicD and the LSNon-mosaic is shown in figure S2 (b). Keff is surmised using the relation,$K_{eff}=\mu{}_{0}H_{C}M_{S}/2$, where HC and MS are coercivity and saturation magnetization of LSFO/SRO bilayers, respectively. Keff for LSMosaicC is slightly larger than the LSNon-mosaic in the entire temperature range.S2 But, with subsequent training Keff is lowered in the LSMosaicD. Furthermore, the Keff of LSMosaicC, LSMoaicD and LSNon-mosaic decreases monotonically with increasing temperature, and exhibits nearly the same trend as that observed for HEB and HC.

**References:**

1. X. K. Ning, Z. J. Wang, X. G. Zhao, C. W. Shih, and Z. D. Zhang. Exchange bias in La0.7Sr0.3MnO3/NiO and LaMnO3/NiO interfaces. *J. Appl. Phys.* **113**, 223903 (2013).
2. Ralph Skomski and J. M. D. Coey. Giant energy product in nanostructured two-phase magnets. *Phys. Rev. B* **48**, 15812 (1993).
